# Highly Parallel Genome-Wide Expression Analysis of Single Mammalian Cells

**DOI:** 10.1371/journal.pone.0030794

**Published:** 2012-02-08

**Authors:** Jian-Bing Fan, Jing Chen, Craig S. April, Jeffrey S. Fisher, Brandy Klotzle, Marina Bibikova, Fiona Kaper, Mostafa Ronaghi, Sten Linnarsson, Takayo Ota, Jeremy Chien, Louise C. Laurent, Sean V. Nisperos, Gina Y. Chen, Jiang F. Zhong

**Affiliations:** 1 Research and Development, Illumina, Inc., San Diego, California, United States of America; 2 Department of Medical Biochemistry and Biophysics, Karolinska Institutet, Stockholm, Sweden; 3 Department of Experimental Pathology, Mayo Clinic, Rochester, Minnesota, United States of America; 4 Department of Reproductive Medicine, University of California San Diego, San Diego, California, United States of America; 5 Center for Regenerative Medicine, The Scripps Research Institute, La Jolla, California, United States of America; 6 Department of Pathology, University of Southern California, Los Angeles, California, United States of America; Institut Jacques Monod, France

## Abstract

**Background:**

We have developed a high-throughput amplification method for generating robust gene expression profiles using single cell or low RNA inputs.

**Methodology/Principal Findings:**

The method uses tagged priming and template-switching, resulting in the incorporation of universal PCR priming sites at both ends of the synthesized cDNA for global PCR amplification. Coupled with a whole-genome gene expression microarray platform, we routinely obtain expression correlation values of R^2^∼0.76–0.80 between individual cells and R^2^∼0.69 between 50 pg total RNA replicates. Expression profiles generated from single cells or 50 pg total RNA correlate well with that generated with higher input (1 ng total RNA) (R^2^∼0.80). Also, the assay is sufficiently sensitive to detect, in a single cell, approximately 63% of the number of genes detected with 1 ng input, with approximately 97% of the genes detected in the single-cell input also detected in the higher input.

**Conclusions/Significance:**

In summary, our method facilitates whole-genome gene expression profiling in contexts where starting material is extremely limiting, particularly in areas such as the study of progenitor cells in early development and tumor stem cell biology.

## Introduction

Recently, there has been growing interest in obtaining gene expression profiles from single cells, as it has become increasingly evident that the heterogeneity present in cell populations is such that population-based transcriptional profiles may not reflect the regulatory networks functional at the individual cell level [1], [2]. Applications for single cell gene expression profiling include lineage determination in early development and organogenesis, including embryogenesis [3], [4], neuronal [5]–[8] and glial [9] cell differentiation, hematopoietic [10], [11], bone marrow stromal [12], epidermal [13], heart [14], [15] and pancreatic [16] stem cell biology. Apart from facilitating cell lineage mapping an additional key utility of single cell transcriptomics is in clinical diagnostics, particularly the identification of gene expression signatures in circulating tumor cells for use as prognostic markers for metastatic tumors [17] and treatment response [18].

The analysis of single cancer cells can potentially overcome the shortcomings of tumor heterogeneity and help pinpoint driver mutations that spur the initial development of tumors, and identify which mutations lead to metastasis, cancer progression and resistance to therapy. However, a key technological challenge in the transcriptional profiling of single cells is that most whole-genome amplification protocols suffer from significant amplification bias. While there have been several recent advancements in the capture and isolation of single cells, such as cell picking [19], [20] and microfluidic [1], [17], [21] devices, there remains a need for the development of high-throughput, whole-genome gene expression assays for single cells. Example of previously reported assays aimed at attempting to overcome the limitation of single cell or near single cell quantities of starting material [for reviews see [4], [22], [23]] include terminal continuation [24], homomeric tailing [3], [10], Ribo-SPIA technology (Ovation Pico WTA and WT-Ovation One-Direct Amplification Systems) [25], [26], TransPlex Whole Transcriptome Amplification technology (Pico Profiling) [27], template switching [28], [29], multiple displacement amplification (total transcript amplification [30]) and linear antisense RNA amplification [6], [8].

The underlying RNA or cDNA amplification strategies employed in most of these studies include either linear antisense RNA amplification or homomeric/TdT tailing followed by exponential amplification. While the former approach has been a mainstay for amplifying nanogram amounts of total RNA, there have been relatively few studies in which single cell quantities have been assayed [6], [8]. Reported disadvantages to this approach include inefficiencies during second strand cDNA synthesis and purification [31], a multi-day workflow [32], time-dependent RNA degradation [33], as well as transcript representation bias [34] all of which are associated with successive rounds of amplification. Variations of the latter approach include A- [3], [4] or G-tailing [10] in order to tag the 3′ end of the first cDNA strand for global PCR amplification. A third strategy by which cDNA may be tagged makes use of a reverse transcriptase with terminal transferase activity facilitating template-switching [20], [28], [35], [36]. Other options by which the 3′ termini of cDNAs may be tagged, include linker/adaptor ligation [37] or the use of a terminal-tagging oligo (TTO) [38]. The linker/adaptor ligation protocol generally requires several additional enzymatic and washing steps, and is therefore not only prone to loss of material, but also cross-contamination. Because both of these methods currently require nanogram quantities of total RNA as inputs, it is likely that the efficiency with which mRNAs are converted into tagged and amplifiable cDNA templates is lower than either the template-switch or homomeric/TdT tailing methods. Recently, a ϕ29 DNA polymerase-based multiple displacement amplification method was described in which the transcriptomes of single bacterial cells were profiled, yielding assay reproducibilities of *R^2^*∼0.80 [30]. While, currently this isothermal technology is adapted for prokaryotes, the authors suggest that it may be modified for use within a eukaryotic context.

Many of these approaches have not been widely adopted either because they suffer from amplification bias, are not sufficiently scalable or robust for high-throughput applications, are not suitable in eukaryotic contexts, or a combination of these factors. Here we describe a template-switch-based high-throughput method that is capable of generating robust whole-genome gene expression profiles at the single cell level.

## Results

**Figure 1:**
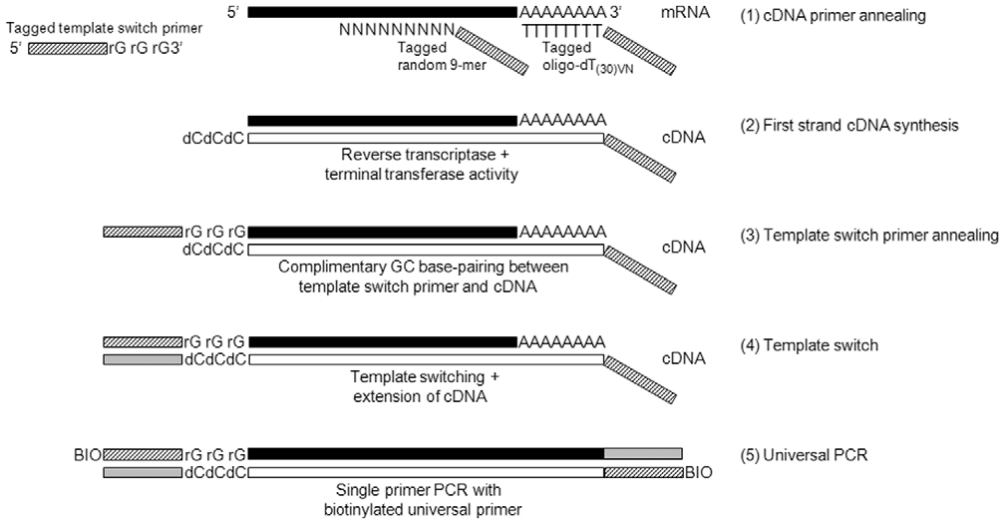
Pre-amplification scheme. (1) First strand cDNA synthesis is primed with tagged oligo-dT and random 9-mer primers. The tagged oligo-dT primer contains a VN anchor followed by a T-30 stretch with a 5′ PCR tag. The tagged random 9-mer consists of a 9-mer followed by the identical 5′ PCR tag. (2) Upon reaching the 5′ terminus of the mRNAs, the reverse transcriptase, via its terminal transferase activity, adds a few nucleotides (predominantly deoxycytidine) to the 3′ end of the newly synthesized cDNAs. (3) The template-switch primer, which consists of the same 5′ PCR tag as well as a 3′ riboguanine stretch, anneals via GC complimentary base-pairing to the 3′ end of the cDNAs, thereby serving as a new template for the reverse transcriptase. (4) After cDNA synthesis, both ends of the cDNAs now contain the identical PCR tag, allowing exponential amplification of the entire cDNA population through single primer PCR (5).

The pre-amplification method described here exploits the template switching ability of some reverse transcriptases which allows the 3′ tagging of cDNA, thereby facilitating the incorporation of universal PCR primer sites at both ends of the synthesized cDNAs (Fig. 1). Here we report on the comprehensive characterization of the performance of our single cell gene expression assay, termed Whole-Genome Gene Expression in Single Cells (WG-XSC), using picogram quantities of total RNAs, as well as a variety of different single cell types. We describe the utility of the WG-XSC assay in the transcriptional profiling of single cells and low input material, for which existing conventional methods are not sufficiently sensitive.

### Pre-Amplification Assay Optimization

Previous template-switching-based amplification protocols utilized oligo-dT-based priming for cDNA synthesis followed by a single-phase PCR amplification reaction [20], [28], [35], [36]. We made several modifications to these specific steps that led to substantial improvements in both the cDNA yield and representation of single cell quantities of starting material. These improvements are summarized in Table 1.

**Table 1:** Values are averages for at least two technical replicates.

Table 1. Optimization of Preamplification Assay					
Input	Condition	Self-Reproducibility (R^2^)	Correlation with 1 ng (R^2^)	Sensitivity^a^	Probe Concordance (%)^b^
50 pg UHR total RNA^c^	T30 + one-phase PCR	0.374	0.473	6595	95.9
50 pg UHR total RNA^c^	T30 + two-phase PCR	0.481	0.585	8019	95.1
50 pg H9 total RNA^c^	T30 + N6 + one-phase PCR	0.626	0.695	10449	92.4
50 pg H9 total RNA^c^	T30 + N9 + one-phase PCR	0.627	0.688	10332	92.2
50 pg UHR total RNA^d^	T30 + N9 + two-phase PCR	0.698	0.806	13443	96.6
Single HeLa cells^d^	T30 + N9 + two-phase PCR	0.757	0.801	11083	97.4

Values shown for the self-reproducibility and correlation are derived from all probes.

a Sensitivity is calculated as the number of probes detected at p-value<0.01.

b Probe concordance is calculated as a percentage of the number of probes with matching detected calls at p-value<0.01 between the low (50 pg or single cell) and standard (1 ng) inputs divided by the total number of probes detected in the lower input.

c 24 K WG-DASL.

d 29 K WG-DASL HT.

We first assessed the impact of different cDNA priming methods, during the reverse transcriptase step, on the performance of our assay. Here we evaluated three conditions, namely: oligo-dT (T30), oligo-dT + random hexamer (T30+N6) or oligo-dT + random nonamer (T30+N9). Replicate inputs of 50 pg H9 cell total RNA were used for all tested priming conditions after which pre-amplified products were used as inputs for the 24 K WG-DASL Assay. While typical assay reproducibilities of R^2^∼0.37 were obtained for the T30 condition, improved self-correlations of R^2^∼0.63 were observed for both the T30+N6 and the T30+N9 priming conditions (Table 1). We also obtained a concomitant 58% increase in the assay sensitivity with the T30 + randomer priming conditions yielding approximately 10449 and 10332 probes detected (p<0.01) for the T30+N6 and T30+N9 conditions, respectively, compared with 6595 detected probes (p<0.01) for the T30 condition (Table 1). Moreover, raw intensity correlations of the lower 50 pg with the higher 1 ng input yielded R^2^∼0.69 for both the T30+N6 and the T30+N9 priming conditions, while yielding R^2^∼0.47 for the T30 condition (Table 1). Our results clearly demonstrate an improvement in the assay performance with the oligo-dT + randomer compared to the oligo-dT priming.

Previous experiments performed with different numbers of PCR cycles (15, 18, 21, 24 and 27 cycles) using different RNA inputs (50 pg and 1 ng) demonstrated that the assay performance (reproducibility, sensitivity and correlation with higher inputs), was poorest at the extremes of our chosen cycle ranges (15 and 27), but optimal at 21 PCR cycles (data not shown). To reduce the impact of stochastic effects on low copy numbers during the early cycles, we sought to improve the efficiency and fidelity of amplification by applying an altered thermal profile for the first few PCR cycles. We next therefore assessed the effect of two different PCR cycling profiles on our assay performance, namely a single-phase profile with an annealing temperature of 65°C, and a 24 cycle, two-phase profile consisting of an initial five PCR cycles carried out at a lower annealing temperature (58°C), followed by 19 cycles at a higher (65°C) annealing temperature (see Materials and Methods for details). For this experiment, replicate inputs of 50 pg UHR total RNA were processed using the 24 K WG-DASL Assay. Typical results showed superior performance using the two-phase condition, as assessed by measures of reproducibility (from R^2^∼0.37 for the one-phase and R^2^∼0.48 for the two-phase conditions) and sensitivity (∼6595 probes detected for the one-phase and 8019 probes detected for the two-phase conditions, p<0.01) (Table 1). This corresponded to a 22% higher assay sensitivity for the two-phase profile. Comparing raw correlations between lower 50 pg input RNA amounts to higher 1 ng inputs, the one-phase and two-phase PCR conditions yielded R^2^∼0.47 and R^2^∼0.59, respectively (Table 1). Together these data demonstrate an improved performance of the two-phase condition as compared with the one-phase profile.

### Performance with RNA Inputs and Single Cells

**Figure 2:**
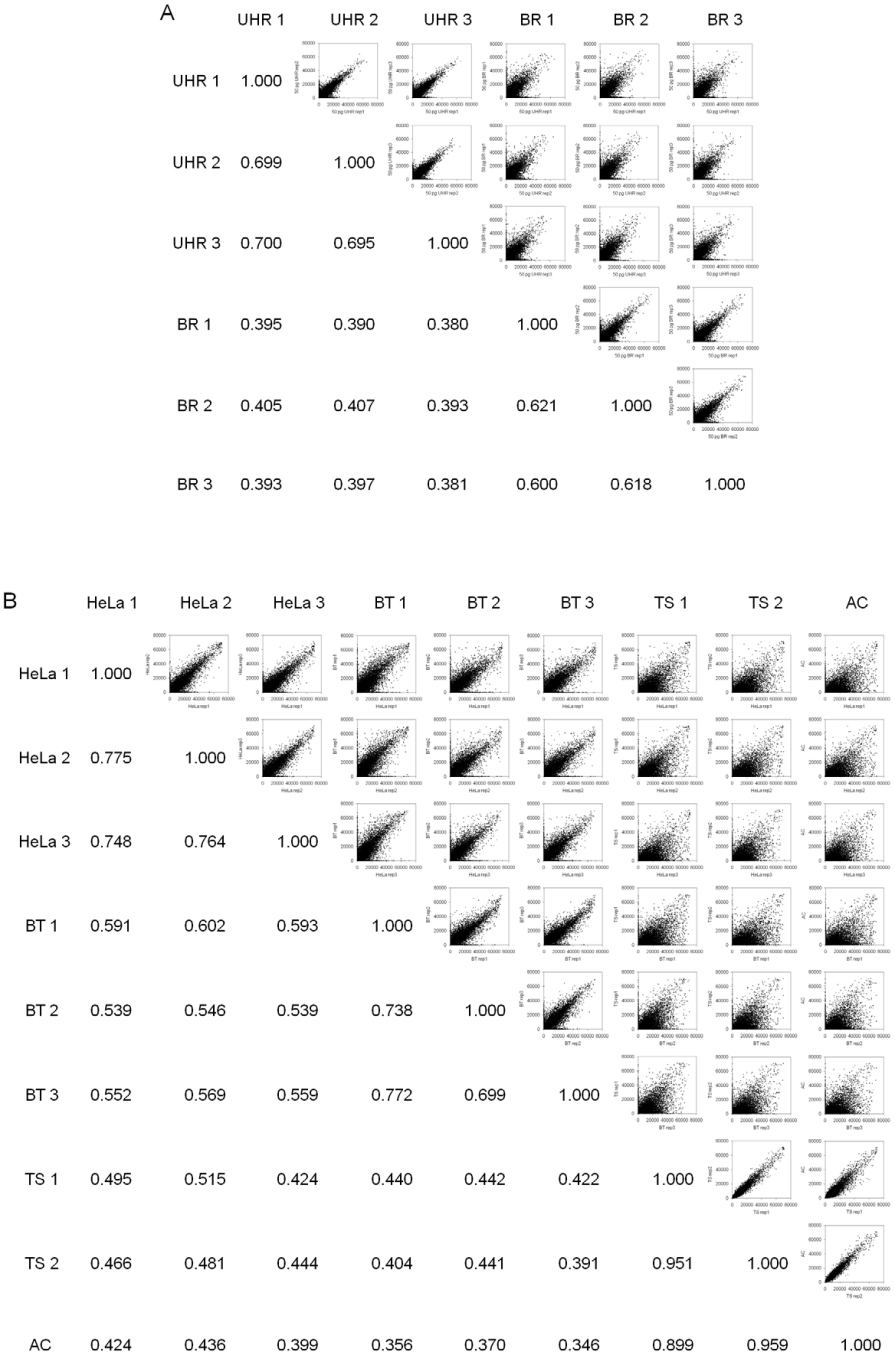
Raw signal intensity correlations between replicates of low input RNAs and whole cells. (A) 50 pg UHR and BR total RNA and (B) single HeLa and brain tumor (BT) cells; 50 cell tumorsphere (TS) and adherent cells (AC). Pair-wise scatterplots of at least two replicates for each input type are shown for all 29 K probes across the full range of raw signal intensities. Correlations are the square of Pearson's correlation coefficient.

A key performance characteristic of any single cell genomics assay is its ability to discriminate among different samples at low input levels. In order to further characterize our assay we used T30+N9 priming together with the two-phase PCR profile described earlier to assay two different RNA inputs. Triplicate aliquots of UHR and BR, each at 10 pg, 50 pg and 1 ng total RNA were used in conjunction with the 29 K WG-DASL HT Assay. RNA quality was assessed using the Bioanalyzer 2100 and yielded RIN values of 9.6 and 9.2 for the UHR and BR samples, respectively (data not shown). On average, our intra-sample self-reproducibilities were R^2^∼0.42, R^2^∼0.69 and R^2^∼0.96 for the 10 pg, 50 pg and 1 ng UHR and R^2^∼0.34, R^2^∼0.61 and R^2^∼0.95 for the 10 pg, 50 pg and 1 ng BR RNA inputs, respectively (Fig. 2A, Table 1). By contrast, comparisons between the UHR and BR RNA samples, at the 50 pg input level, yielded inter-sample correlations of R^2^∼0.39 (Fig. 2A), whereas UHR vs. BR inter-sample correlations at the 1 ng input level yielded R^2^∼0.61, suggesting that, based on gene expression profiles, our assay can reliably differentiate between different low RNA inputs.

Having obtained robust data using picogram quantities of RNA, we next repeated the experiment, using individual cells as inputs. Here we used single HeLa and primary brain tumor (BT) cells. As before, all samples were processed in triplicate. We observed a similar trend to that obtained for the RNA equivalent inputs, with intra-sample self-reproducibilities of R^2^∼0.76 (Fig. 2B, Table 1) and R^2^∼0.74 for the HeLa and BT samples, respectively, while the inter-sample correlations between the HeLa and BT single cell samples were lower with R^2^∼0.57 (Fig. 2B). The differences between the intra- and inter-sample average correlations were statistically significant as reported by Student's *t*-test (intra-sample HeLa R^2^ vs. inter-sample HeLa-BT R^2^, p = 1.34E-7; intra-sample BT R^2^ vs. inter-sample HeLa-BT R^2^, p = 2.80E-6). Apart from different single cells, we also profiled 5-cell (HeLa) inputs, and obtained self-correlations of R^2^∼0.88. We also compared the single cell HeLa and BT expression profiles with that obtained for 50-cell inputs for tumorspheres (TS) and their adherent cell (AC) counterparts. While the intra-sample correlations for the TS and AC samples yielded R^2^ of 0.89–0.95 (Fig. 2B), the inter-sample correlations between the TS/AC vs. HeLa and the TS/AC vs. BT cells yielded R^2^ of ∼0.45 and ∼0.40, respectively. Together these results indicate that our whole-genome gene expression assay can robustly discriminate among different individual cell types.

We next ranked the fold-change differences between the TS and the AC samples and further analyzed the top 100 over-expressed and 100 under-expressed genes in the tumorspheres relative to their attached counterpart. Using DAVID [39] we extracted GO terms and compared the relative frequencies of these terms in each list to that in the human genome to identify statistically significant enriched GO terms. Within the over-expressed gene list several transcription factors such as SMAD2, SHOC2 and KLHL7 were included under the enriched GO term “nucleus” (p = 8.71E-3) consistent with a stem cell (Fig. S1) versus a more differentiated cellular phenotype. Conversely, for the under-expressed genes over-represented GO terms included “cell/biological adhesion” (p = 3.83E-5) as well as “extracellular space” (p = 5.34E-3) suggestive of a cell-matrix adhesion pathway for an attached (Fig. S1) as opposed to a suspended cell culture.

**Figure 3:**
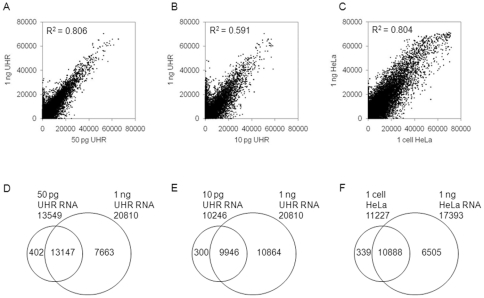
Intensity and detected probe concordance comparisons between low and higher 1 ng inputs. Raw signal intensity correlations between (A) 50 pg (x-axis) and 1 ng (y-axis) UHR total RNA; (B) 10 pg (x-axis) and 1 ng (y-axis) UHR total RNA; (C) single HeLa cell (x-axis) and 1 ng (y-axis) HeLa total RNA. The overlapping sets of detected probes between the low and higher inputs are shown for both the RNA equivalent (D, E) and single cell (F) inputs. All probe values shown are at a threshold of p<0.01.

In order to determine the extent to which the gene expression profiles obtained at low input levels correlated with those obtained with higher inputs, we directly compared raw signal intensities between the lower and higher inputs. Correlations between 50 pg and 1 ng total RNA typically yielded R^2^∼0.80 (Fig. 3A, Table 1), whereas correlations between 10 pg and 1 ng total RNA typically yielded R^2^∼0.59 (Fig. 3B). Single cell correlations with 1 ng total RNA, derived from a corresponding bulk cell culture, yielded R^2^∼0.80 (Fig. 3C, Table 1). At p<0.01, we detected ∼13443 and 10180 probes for the 50 and 10 pg RNA inputs, respectively, whilst detecting ∼14156 and 11083 probes for the 5-cell and single cell inputs (Table 1), respectively. This level of sensitivity represents approximately 77% (50 pg), 58% (10 pg), 80% (5-cell) and 63% (single cell) of the total number of probes detected in the higher, standard inputs. Furthermore, when the lists of probes detected (p<0.01) in the lower inputs were intersected with those detected in the higher 1 ng inputs, we obtained probe concordance values of ∼96.6%, 97.1% and 97.4% for the 50 pg (Fig. 3D, Table 1), 10 pg (Fig. 3E) and single HeLa cells (Fig. 3F, Table 1), respectively. The percentage of false positive probes detected in the lower inputs was <2% of the total number of probes detected in the higher standard inputs. Taken together these results demonstrate that our assay is sufficiently sensitive to reliably detect, in low inputs, most of the genes that are detected at standard higher inputs, and that the expression profiles derived from these lower inputs accurately recapitulate those obtained in higher inputs.

## Discussion

Over the last few years there have been several reported studies on either single cell gene expressing profiling using low gene density (1–100) assays [1], [2], [16], [40]–[42], or inputs of small populations of cells (10–150) [27], [43] or 100 pg–1 ng total RNA [44]–[46] at the whole transcriptome level. Very few genome-wide studies have been reported in which the assay performance has been rigorously characterized using whole single cells or RNA equivalents as inputs [3], [4], [10], [47].

Our WG-XSC assay is highly reproducible, typically yielding R^2^∼0.76 and ∼0.69 for single cell and 50 pg RNA inputs, respectively. The transcript representation as assessed by the correlation between lower inputs and larger standard inputs yielded R^2^∼0.80 and ∼0.81 for single cell and 50 pg RNA inputs, respectively. Of the few microarray-based single cell transcriptional studies with self-correlation metrics the reported R values range between 0.73–0.91 [3], [10], [48]. Exact comparisons between these studies and the current study is challenging as either the underlying experimental designs differ [10] or the analysis methods are different [3], [10], [48]. A recent mRNA-Seq study in which single mouse oocytes were assayed, reported assay reproducibilities of R^2^∼0.97 [4]. It should be noted however that these cells are atypical in size and therefore are also not directly comparable with the current study.

Two obvious, but critical steps that could impact levels of reproducibility and representation include the extent of cell lysis as well as the efficiency with which low abundance mRNA molecules are converted to cDNA. In order to minimize the loss of material, and maximize the synthesis of cDNA in an unbiased fashion, our protocol specifically incorporates the use of a phase-switch microfluidics device and low-retention plasticware for single cell isolation, oligo-dT and random priming for cDNA synthesis and a two-phase thermal profile for PCR amplification.

An additional feature of our approach is the ability to process up to 96 samples in parallel, thereby greatly reducing the associated labor costs as well as minimizing variation/bias that may arise from handling individual samples. This feature is of particular relevance for single cell expression profiling where substantial variation in transcript levels among phenotypically identical single cells has been well documented, thereby necessitating the simultaneous analyses of large numbers of individual cells [2], [20]. In summary, our method facilitates whole-genome gene expression profiling in contexts where starting material is extremely limiting, particularly in areas such as the study of progenitor cells in early development and tumor stem cell biology.

Our high-throughput assay generates whole-genome gene expression profiles with single cell or low RNA inputs. This robust and scalable method for profiling a variety of cell types at the single cell level can be applied to critical questions in a broad range of areas, including developmental biology and cancer biology. We have used the technology for gene expression profiling in circulating tumor cells isolated from prostate cancer and ovarian cancer patients' blood, as well as molecular and functional characterization of early lineage commitment of human hematopoietic stem cells (data not shown). The ability to obtain genome-wide gene expression data on many individual cells in parallel will be extremely valuable in a variety of contexts, including detailed molecular lineage tracing studies and clinical studies aimed at biomarker discovery.

## Materials and Methods

### RNA Extraction

RNA from the WA09 (H9) [49] human embryonic stem cell line was extracted using TRIzol (Life Technologies/Invitrogen, Carlsbad, CA, USA) according to the manufacturer's instructions after which the precipitated RNA pellet was resuspended in 10 µl RNase-free water. Commercial RNAs were purchased from the following vendors, FirstChoice Human Cervical Adenocarcinoma (HeLa-S3) and FirstChoice Human Brain Reference (BR) (both from Life Technologies/Ambion, Austin, TX, USA) and Universal Human Reference (UHR) (Agilent/Stratagene, Santa Clara, CA, USA). Different inputs, as indicated in the Results section, were used for each pre-amplification reaction.

### Sorting of Cultured Cells with a Phase-Switch Microfluidics Device

A microfluidics device with a phase-switch feature was used for isolating individual cells. Briefly, cultured cells were harvested with trypsinization and washed with PBS, whereafter a single cell suspension in PBS was load into a phase-switch microfluidics device for encapsulation of individual cells into droplets. Cells were encapsulated from the aqueous phase (PBS) into droplets in the oil phase by either laser-cavitation or T-junction break-up of immiscible threads as previously described [50]. With optimized parameters, each droplet contained only one single HeLa.S-Fucci (RIKEN BioResource Center Cell Bank, Ibaraki, Japan) [51] or brain tumor (BT) cell. This was visually confirmed under a fluorescent microscope. This approach minimizes the stress on sorted cells and facilitates the manipulation of single cells in droplets. Individual sorted cells were aliquoted in ≤1 µl Single cell Lysis Buffer (SLB, Illumina, Inc.).

### Culture of Tumorspheres and Adherent Cells

An ovarian cancer cell line, RMG1 [52] was cultured in three T-150 flasks until 80–90% confluency in M199/MCDB105 medium (Sigma, St. Louis, MO, USA) supplemented with 5% FBS (HyClone Laboratories Ltd., Logan, UT, USA), penicillin (100 U/ml), and streptomycin (100 µg/ml). To culture under stem cell conditions [53] cells were trypsinized and then resuspended in DMEM/F12 medium supplemented with 5 µg/ml insulin (Novo Nordisk Inc., Princeton, NJ, USA), 20 ng/ml epidermal growth factor (R&D Systems, Inc., Minneapolis, MN, USA), 10 ng/ml basic fibroblast growth factor (Invitrogen, Carlsbad, CA, USA), 0.4% bovine serum albumin (BD Falcon; Bedford, MA, USA), penicillin (100 U/ml), and streptomycin (100 µg/ml) in two 100 mm Ultra Low Attachment plates (Corning, Lowell, MA, USA) over three weeks. Spheroids were selected using a 40 µm cell strainer (BD Falcon, Bedford, MA, USA), after which half of the spheroids were cultured in one 35 mm dish (adherent cells, AC) and the other half were grown in one well of one 6 well low attachment plate in stem cell medium (tumorspheres, TS) for two days. Cells were grown at 37°C in a 5% CO_2_/air atmosphere. The brain tumor (BT) cells, derived from U118 human glioblastoma cells (ATCC HTB-15), were purchased from the ATCC (Rockville, MD, USA) and maintained in DMEM supplemented with 10% fetal bovine serum, glutamine, and penicillin/streptomycin as recommended by the ATCC.

### Cell Lysis, cDNA Synthesis and Pre-Amplification

All cell lysis and cDNA reactions were performed using 0.2 ml Maxymum Recovery PCR tubes (Axygen, Union City, CA, USA). The cell lysis, reverse transcription, template switching and pre-amplification reactions were all performed in a single tube. Briefly, for cell lysis, 1.8 µl SLB was added directly to the isolated single cell. Tubes were placed in a thermocycler and heated to 72°C for 3 min, followed by five min at 4°C. After cell lysis, 3.2 µl Single cell cDNA Synthesis Buffer (SCB, Illumina, Inc.) was added to the lysed single cell. The reverse transcription and template switching reactions were performed at 42°C for 60 min, followed by a 10 min 70°C inactivation step. After cDNA synthesis 32 µl of Single cell PCR Mix (SPM, Illumina, Inc.) was added directly to the unpurified products followed by amplification using a PCR cycling profile which consisted of an initial denaturation of 95°C for 1 min, followed by 5 cycles of (95°C for 20 sec 58°C for 30 sec and 68°C for 3 min), 9 cycles of (95°C for 20 sec, 65°C for 30 sec and 68°C for 3 min), 10 cycles of (95°C for 30 sec, 65°C for 30 sec and 68°C for 3 min+6 sec/cycle) and 1 cycle of 72°C for 10 min. For cell-equivalent RNA inputs, the SLB and SCB were added directly to the RNA (the cell lysis step was omitted) and were reverse-transcribed and pre-amplified in the identical manner to that described for the cell lysates.

### Whole-Genome DASL® Assay

For whole-genome gene expression analysis, we used either the Whole-Genome DASL Assay or the Whole-Genome DASL HT Assay, an updated version of the original Whole-Genome DASL Assay [54]. Briefly, the WG-DASL assay probes ∼24 K targets (∼18 K unique genes) and uses the HumanRef-8 v3 BeadChip, while the WG-DASL HT Assay interrogates ∼29 K targets (∼21 K unique genes), based on content derived from the National Center for Biotechnology Information Reference Sequence Database (Release 98, November 2009) and uses the Human HT-12 v4 BeadChip. For both 24 K and 29 K assays we used 10 µl of the 37 µl (27%) pre-amplified cDNA product which we annealed directly to the 24 K or 29 K oligo pool and then proceeded exactly as previously described [54].

### Microarray Data Analysis

Unless otherwise stated, all data were analyzed in an un-normalized, raw state. All individual samples were assayed a minimum of two times. After scanning, intensity data were imported into GenomeStudio® v2.0 where the data quality was assessed using several assay controls. Detection p-values were computed using several hundred negative controls to determine gene expression detection limits. Assay performance metrics are described further in the Results section. All of the microarray data are MIAME-compliant (http://www.mged.org/Workgroups/MIAME/miame.html) and have been submitted to GEO (Accession Number: GSE34365). Over-representation analysis of differentially expressed genes was performed using DAVID [39], which reports functional gene categories as statistically significant gene ontology (GO) terms.

## Supporting Information

Figure S1 **Ovarian cancer cells (RMG1) were cultured in stem cell media.** (A) a spheroid in an ultra-low attachment plate, (B) a spheroid in a tissue-culture treated plate, after two days in a regular plate. Scale bar = 90 µm.
(PDF)
