# Correction: Highly Parallel Genome-Wide Expression Analysis of Single Mammalian Cells

**DOI:** 10.1371/annotation/3f0ddf41-2e7a-4b56-93c2-2731f2890b19

**Published:** 2012-02-21

**Authors:** Jian-Bing Fan, Jing Chen, Craig S. April, Jeffrey S. Fisher, Brandy Klotzle, Marina Bibikova, Fiona Kaper, Mostafa Ronaghi, Sten Linnarsson, Takayo Ota, Jeremy Chien, Louise C. Laurent, Jeanne F. Loring, Sean V. Nisperos, Gina Y. Chen, Jiang F. Zhong

The 13th author was omitted. The 13th author is Jeanne F. Loring. Dr. Loring's affiliation is: 5 Center for Regenerative Medicine, The Scripps Research Institute, La Jolla, California, United States of America. The author's contributions are: Conceived and designed the experiments and Contributed reagents/materials/analysis tools. 

