# Supplementary material for: Highly Parallel Genome-Wide Expression Analysis of Single Mammalian Cells
Source: PLoS One. 2012 Feb 8;7(2):e30794. doi: 10.1371/journal.pone.0030794 (PMC3275609; doi:10.1371/journal.pone.0030794)
Supplement: Figure S1 — Ovarian cancer cells (RMG1) were cultured in stem cell media. (A) a spheroid in an ultra-low attachment plate, (B) a spheroid in a tissue-culture treated plate, after two days in a regular plate. Scale bar = 90 µm. (PDF) [file pone.0030794.s001.pdf]

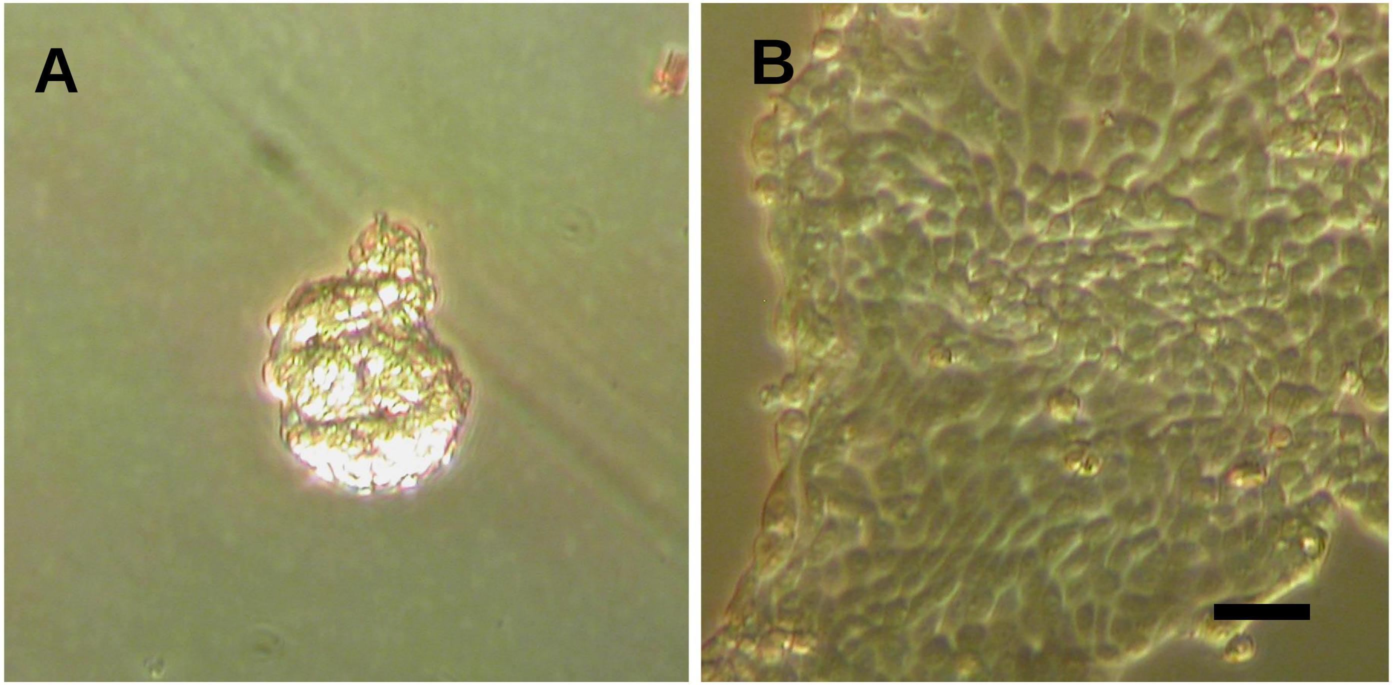

Figure S1. Ovarian cancer cells (RMG1) were cultured in stem cell media. (A) a spheroid in an ultra-low attachment plate, (B) a spheroid in a tissue-culture treated plate, after two days in a regular plate.
Scale bar = 90 μm
